# Mitigation of salt stress in Indian mustard (*Brassica juncea* L.) by the application of triacontanol and hydrogen sulfide

**DOI:** 10.1080/15592324.2023.2189371

**Published:** 2023-03-19

**Authors:** Tunisha Verma, Savita Bhardwaj, Ali Raza, Ivica Djalovic, PV Vara Prasad, Dhriti Kapoor

**Affiliations:** a Department of Botany, School of Bioengineering and Biosciences, Lovely Professional University, Phagwara, India; b College of Agriculture, Fujian Agriculture and Forestry University (FAFU), Fuzhou, China; c Institute of Field and Vegetable Crops, National Institute of the Republic of Serbia, Novi Sad, Serbia; d Department of Agronomy, Kansas State University, Manhattan, KS, USA

**Keywords:** Abiotic stress, antioxidants, climate change, gaseous molecule, metabolites, plant growth regulators

## Abstract

Salinity stress is a well-known abiotic stress that has been shown to have a negative impact on crop growth, production, and soil richness. The current study was intended to ameliorate salt stress in Indian mustard (*Brassica juncea* L.), keeping in mind the detrimental influence of salt stress. A pot experimentation was executed on *B. juncea* to examine the efficacy of exogenous application of triacontanol (TRIA) and hydrogen sulfide (H_2_S) (NaHS donor), either alone or in combination, on growth attributes, metabolites, and antioxidant defense system exposed to salt stress at three distinct concentrations (50, 100 and 150 mM NaCl). Increase in the concentration of oxidative markers (malondialdehyde and hydrogen peroxide) was found which results in inhibited growth of *B. juncea*. The growth characteristics of plant, such as root and shoot length, fresh and dry weight under salt stress, were improved by foliar application of TRIA (150 µM) and H_2_S (25 µM) alone as well as in combination. Additionally, salt stress reduced the levels of protein, metabolites (flavonoids, phenolic and anthocyanin), antioxidant enzyme activity including that of ascorbate peroxidase, catalase, polyphenol oxidase and guaiacol peroxidase as well as the level of ascorbic acid and glutathione (non-enzymatic antioxidants). However, application of TRIA and H_2_S alone or in grouping substantially raised the content of protein, metabolites and antioxidant defense system in plants of *B. juncea*.

## Graphical abstract

**Figure:**
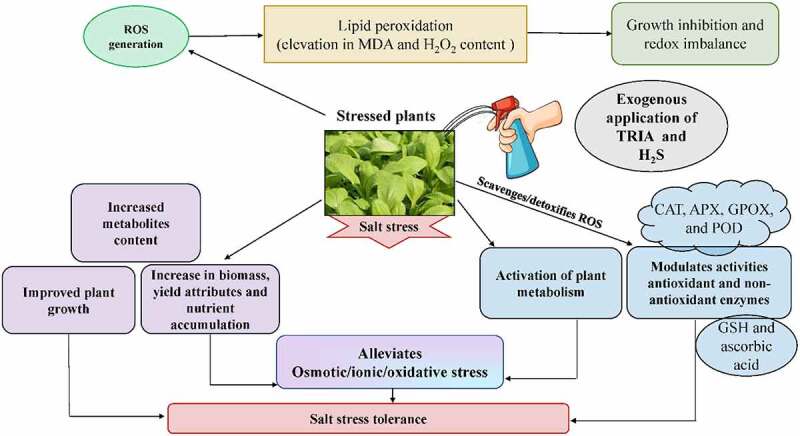

## 1. Introduction

Stress is characterized as a foremost environmental factor that adversely impacts crop growth, development, and efficiency. Due to current and upcoming climate change events, plants experience numerous abiotic (drought, extreme temperature, osmotic, and salinity) and biotic stresses (insect, pathogen, and herbivore) during their life cycle.^1,2^ Out of all the environmental stresses, salt stress limits agricultural growth and productivity globally.^3^ Salinity is a widespread global concern caused as a result of the wrong usage of agricultural lands, excess evaporation, and lack of drainage. The continuous increase in salinity has devastating global effects. It has been estimated that approximately 6% of the area is prone to salt stress, and this level is rising day by day due to expected and human caused events.^3,4^ Several studies pertaining to salt stress have described a reduction in growth of plants as a result of an upsurge in toxicity of specific ions (Na^+^ and Cl^−^ ions), oxidative stress, hormonal imbalance, interference in nutrient uptake, the disturbed relationship between leaf and water, membrane disorganization and resulting in oxidative stress due to formation of reactive oxygen species (ROS).^3,5,6^ In order to deal with the negative impacts of salt stress, plants undergo different morpho-physiological, molecular, cellular, and anatomical changes.^3,7,8^ Therefore, it is important to apply novel stress tolerance strategies to cope with the adverse effects of stresses such as soil salinity. One good and effective way to help plants deal with stress is to use plant growth regulators (PGRs), which help plants do their functional normally.^9–16^

Phytohormones are chemical molecules that are formed in deficient concentrations and play a key role in the mitigation of several abiotic stresses by regulating various metabolic processes in plants.^12^ They work as chemical messengers which regulate growth and development by coordinating signal transduction pathways in plants.^9–12^ The role of some phytohormones, such as auxin, ABA, gibberellins, ethylene, and salicylic acid in salinity tolerance is well-defined.^9–11^ However, they differ in their mode of action. These PGRs are well-known to play an essential role in modulating a variety of activities in plants.^17^ Triacontanol (TRIA) has emerged as new PGR that adjusts multiple physio-biochemical activities in plants and helps in reducing the salinity stress in plants. TRIA is a long chain of 30-carbon primary alcohol which promotes plant growth and output via exogenous application.^18^ This PGR has attracted the interest of researchers due of its regulatory effect on photosynthesis, water and nutrient assimilation, antioxidant enzyme activities, membrane stability, and gene regulations. As an useful PGR, TRIA has been used in different plant species, which encourages root-shoot development and produces secondary metabolites.^19–25^ Numerous findings have revealed the importance of TRIA in boosting plant growth, biomass, photosynthetic pigment, and regulation of vital nutrients under saline conditions.

Different substances, including phytohormones and signaling molecules, interact with one another to cope up with diverse stresses in response to changing environmental conditions. Signaling molecules are known for plant growth, development, and adaptation and for activating different antioxidant responses to different forms of stresses.^12^ Recently, hydrogen sulfide (H_2_S) has become known as a novel signaling gaseous molecule that elicits several stress responses. H_2_S is a colorless gaseous plant signaling molecule that plays a essential role in mediating plant growth and improvement under both normal physiological as well as biotic and abiotic conditions.^13,26^ Exogenous applications of H_2_S, utilizing different donors such sodium hydrosulfide (NaHS), have been demonstrated to significantly reduce plant damage, especially by activating antioxidant enzymatic and non-enzymatic mechanisms, according to a growing number of studies.^13,26^ H_2_S is a major signaling molecule that regulates numerous aspects of plant development and growth, including photosynthesis, stomatal movement, and seed germination.^21,27,28^ H_2_S also helps to mitigate the unpleasant outcomes of several abiotic stressors, such as salt, heavy metals, heat, and drought.^13,28,29^

Among the major oilseed species, Indian mustard (*Brassica juncea* L.) is most affected by salinity as it is primarily grown in arid and semiarid regions. *B. juncea* belongs to the family *Brassicaceae*. It is an annual herb that is widely cultivated because of its edible and therapeutic properties. *B. juncea* is rich in dietary fibers, vitamins, minerals, fats, protein and carbohydrates, total sugar content, amino acids, ascorbic acid, and antioxidants. As it is widely renowned for its nutritional and economic importance, *Brassica* occupies third place among the different oilseed species.^29^ However, its growth, yield, and oil production are drastically affected by salinity.^30^ In the last two decades, in view of the growing reorganization of the health benefits of mustard and its present possible salt tolerance, demand for it has undoubtedly increased. A beneficial approach to reducing salt stress in *B. juncea* is required to meet its rising demand for the growing population.^31^

H_2_S as signaling molecule interacts with various PGRs by regulating their anabolism and catabolism, transport and distribution, sulfhydration of their receptors, and other signaling pathways. Similarly, PGRs interact with H_2_S by modulating its anabolism and catabolism by modulating the metabolic enzymatic activity and gene expression. Similar could be the possible ways of interaction between TRIA and H_2_S. Considering the versatile utility of *B. juncea*, dearth of knowledge on TRIA and H_2_S, and the paucity of research on the malicious effect of salt stress, the current research has been proposed to examine the effect of TRIA and H_2_S molecules in mitigating the negative impacts of salinity on growth, physiological, and biochemical traits.

## 2. Materials and methods

### 2.1. Plant materials, growth conditions, and treatments

*B. juncea* seeds (var. PBR-91) were procured from Punjab Agricultural University, Ludhiana, India. The present research was performed under natural environmental conditions at the agricultural farm of Lovely Professional University, Phagwara, Punjab. After being sterilized for five minutes with 1% sodium hypochlorite, the seeds were rinsed three times in distilled water. An effective concentration of TRIA (150 µM) and H_2_S (25 µM) was chosen through a preliminary study, which resulted in a higher plant growth rate.

Seeds of *B. juncea* were pre-treated with 150 µM TRIA for 8 h, same amount of time was taken to dip the remaining seeds in distilled water. Agro-bags (24 cm in diameter and 40 cm in height) containing soil and organic manure (vermicompost) in a 3:1 ratio. In the preliminary experiment, 50% reduction in growth observed at 100 mM NaCl concentration, so the final concentrations chosen for the present study were 50, 100, and 150 mM. An effective concentration of TRIA (150 µM) and H_2_S (25 µM) was chosen based on a higher plant growth rate. Fifteen plants were retained in an agro-bag after the seedling emergence. Exogenous application of H_2_S was given to the plants in the form of NaHS (in liquid form) at 25 µM concentration after 5 days of seedling emergence. Plants were harvested on the 30^th^ day after sowing and various morphological and biochemical parameters were evaluated. Plant leaves were used for further analysis. The experiment included 16 treatments: (1) CN (control), (2) NaCl I (50 mM), (3) NaCl II (100 mM), (4) NaCl III (150 mM), (5) TRIA, (6) TRIA + NaCl I, (7) TRIA + NaCl II, (8) TRIA + NaCl III, (9) H_2_S, (10) H_2_S + NaCl I, (11) H_2_S + NaCl II, (12) H_2_S + NaCl III, (13) TRIA + H_2_S, (14) TRIA + H_2_S + NaCl I, (15) TRIA + H_2_S + NaCl II and (16) TRIA + H_2_S + NaCl III.

### 2.2. Measurement of growth attributes

The 30-day-old plants of *B. juncea* were measured for the growth characteristics, including root length (cm), shoot length (cm), and fresh and dry weight (mg) of complete plants.

### 2.3. Oxidative stress markers

#### 2.3.1. Malondialdehyde content (MDA)

Heath and Packer^32^ method was used to calculate MDA content. To 0.1 g finely grounded plant tissue, 0.1% trichloroacetic acid (TCA) was used to homogenize it and centrifugation of mixture was carried out at 5,000 rpm Reaction mixture was prepared by using 20% TCA with 0.5% thiobarbituric acid (TBA) to 1 mL of supernatant and then heated at 95°C for 30 minutes before chilling on ice. Absorbance of sample was measured using a spectrophotometer at a wavelength of 532 nm, and nonspecific absorbance was corrected by deducting the absorbance taken at 600 nm using 155 mM^−1^ cm^−1^ as an extinction coefficient.

#### 2.3.2. Hydrogen peroxide (H_2_O_2_) content

Method proposed by Velikova et al.^33^ was used to check the H_2_O_2_ content. Then, 100 mg of fresh tissue was used for homogenization with 0.1% of TCA. For 15 minutes, the centrifugation of the reaction mixture was carried out at 12,000 rpm. A UV-visible spectrophotometer was used to measure the absorbance at 390 nm after adding 0.4 mL of potassium phosphate buffer (10 mM, pH 7.0) and 0.8 mL of potassium iodide (1 M, pH 7.4) to 0.5 mL of supernatant. Then, standard curve was plotted to calculate the H_2_O_2_ content.

### 2.4. Metabolites

#### 2.4.1. Flavonoid content

Content of flavonoid was calculated by using method proposed by Kim et al.^34^ Briefly, 500 mg of fresh tissue sample was used for carrying out homogenization absolute methanol of 3 mL followed by centrifugation of homogenate at 13,000 rpm for 20 min. To 1 mL of supernatant, 4 mL of double distilled water (DDW), 0.3 mL of sodium nitrite (NaNO_2_) and 0.3 mL of aluminum chloride (AlCl_3_) were added and then incubation was carried out for 5 min. The pink coloration was observed after the incorporation of 2 mL sodium hydroxide (NaOH) to the 2.4 mL distilled water. The calculation of flavonoid content was done through a standard curve. Rutin was taken as standard and wavelength was set at 510 nm to take optical density.

#### 2.4.2. Phenolic content

Content of phenols was determined using protocol of Malick and Singh.^35^ Fresh leaf tissue of 0.5 g was homogenized in 80% ethanol. Homogenate was then centrifuged for 20 min. at 10,000 rpm. To supernatant of 0.5 mL Folin-Ciocalteau (FC) reagent and 20% Na_2_CO_3_ was added. Extracted samples were read for carrying out at 650 nm, and the phenolic content was estimated using standard as gallic acid.

#### 2.4.3. Anthocyanin content

The protocol by Mancinelli^36^ was used for determining content of anthocyanin. Extraction mixture was formed by mixing methanol: water: HCl in a ratio 79:20:1. Fresh tissue of 0.5 g was crushed in 3 mL of extraction mixture. The centrifugation of homogenate was done for 20 min at 13,000 rpm. Absorbance of the supernatant was read at wavelength of 530 and 657 nm.

### 2.5. Protein content and activities of antioxidative enzymes

#### 2.5.1. Protein content

Estimation of protein content was done by protocol of Lowry.^37^ Extraction mixture was formed by using 500 mg of plant tissue and 3 ml of phosphate buffer and centrifugation was carried out at 10,000 rpm for 10 minutes. Distilled water of 0.9 mL was added to 0.1 mL of supernatant. Then, 5 mL of reagent C was added to each tube, which was a mixture of reagents A and B (copper sulfate in potassium sodium tartrate and sodium carbonate in sodium hydroxide). The reaction mixture was made to stand for 10 min after mixing well. 0.5 mL Reagent D (FC reagent) was added, mixed, and heated for 30 minutes at room temperature in the dark. The blue coloration was developed. Readings were taken at absorbance of 660 nm by using bovine serum albumin (BSA) as standard.

#### 2.5.2. Antioxidative enzyme activities

To appraise the activity of catalase (CAT), the protocol described by Aebi^38^ was used. The extraction mixture was prepared by using 1 g plant sample in 3 mL phosphate buffer (100 mM, pH 7.0) and centrifugation was done at 5,000 rpm for 20 minutes. Change in absorbance was measured at 240 nm by adding 50 µL of plant sample to 300 µL of H_2_O_2_ (150 mM) and 2.650 mL of 100 mM phosphate buffer. The extinction coefficient used was 43.6 mM^−1^cm^−1^.

Nakano and Asada^39^ protocol was used to evaluate enzymatic activity of ascorbate peroxidase (APX). Extraction was carried out in 1 g leaf tissue in 3 mL phosphate buffer (100 mM, pH 7.0) and centrifugation was performed 20 minutes at 5,000 rpm. Then, plant extract of 50 µL was added in the cuvette containing 2.370 mL of phosphate buffer, 0.3 mL ascorbate (5 mM) and 0.3 mL H_2_O_2_ (0.5 mM). Further, absorbance was read at 290 nm using spectrophotometer and extinction coefficient used was 2.8 mM^−1^ cm^−1^.

The standard protocol by Bergmeyer^40^ was used for guaiacol peroxidase (GPOX) enzyme activity. Fresh plant sample (1 g) was used for carrying out extraction in phosphate buffer (100 mM, pH 7.0) of 3 mL and the sample was centrifuged for 20 min at centrifugation was done at 5,000 rpm for 20 minutes. After this, plant sample of 50 µL was added in the cuvette using phosphate buffer of 2.370 mL, 0.3 mL guaiacol and 0.3 mL H_2_O_2_. Spectrophotometer was set at 436 nm for taking absorbance. The extinction coefficient was 25.5 mM^−1^ cm^−1^.

Polyphenol oxidase (PPO) enzymatic activity was determined by using Kumar and PA^41^ method. Then 50 µL of plant sample was added in the cuvette consisting of 1950 µL of phosphate buffer (0.1 M), 0.5 mL catechol (0.1 M), and 2.5 N H_2_SO_4_. Readings were taken at absorbance of 495 nm using spectrophotometer. The extinction co-efficient used was 2.9 mM^−1^ cm^−1^.

### 2.6. Non-enzymatic antioxidants

#### 2.6.1. Ascorbic acid (AsA)

Protocol by Roe and Kuether^42^ was used to evaluate the ascorbic acid content. Briefly, 50 mM tris buffer (pH 10.0) was used to homogenize 0.5 g of fresh plant tissue, carried out by centrifugation for 20 min at 13,000 rpm. The plant extract of 0.5 mL, 100 mg charcoal, 4 mL of DDW, and 50% TCA of 0.5 mL was added, followed by filtration with Whatman filter paper 1. Incubation was done at 37°C at 3 h after adding 0.4 mL of DNPH (2,4-dinitrophenyl hydrazine) and 1.6 mL cold H_2_SO_4_ (65%) was placed to the mixture. Afterward, mixture was kept at room temperature for 30 minutes. Readings were taken at absorbance of 520 nm using spectrophotometer. Ascorbic acid (1 mg 100 mL^−1^) was used as standard.

#### 2.6.2. Glutathione content (GSH)

Content of glutathione content was determined by using the protocol given by Sedlak and Lindsay.^43^ Leaf sample (1 g of each sample) was finely grounded in tris buffer of 3 mL (50 mM, pH 10.0) and centrifugation was carried out for 20 min at 13,000 rpm. Plant extract of 100 µL, 1 mL of Tris buffer (0.2 M, pH 8.2), 50 µL of 0.01 M DTNB [(5,5’-dithiobis-(2-nitrobenzoic acid)] and absolute methanol of 4 mL was added in test tube. Following this, incubation was done for 15 minutes at room temperature and was centrifuged for 15 min at 3,000 rpm. Furthermore, the optical density of the solution was read using a spectrophotometer at 412 nm using a reference of 100 mL^−1^ mg glutathione to determine the glutathione concentration.

### 2.7. Statistical analysis

Present data was performed in SPSS 16.0 software (SPSS Inc., Chicago IL, USA). One-way analysis of variance (ANOVA) was employed to do the statistical analysis, and Tukey’s test with a significance threshold of P < 0.05 was utilized to compare the treatments. Means with dissimilar letters in the bar graphs are statistically significant at a *p* value less than 0.05. Triplicates (three pots per replication) were used for each experiment and performed two times independently. Data is expressed as the means ± SEM in figures. RStudio was used to run the fviz-pca function from the factoextra R package version 1.0.7 to perform principal component analysis (PCA). The mcor function in RStudio was used to perform a Pearson’s correlation analysis, and the corrplot package Ver. 0.89 was used to create corrplots.

## 3. Results

### 3.1. TRIA and H_2_S-mediated plant growth parameters under salt stress

**Figure 1.**
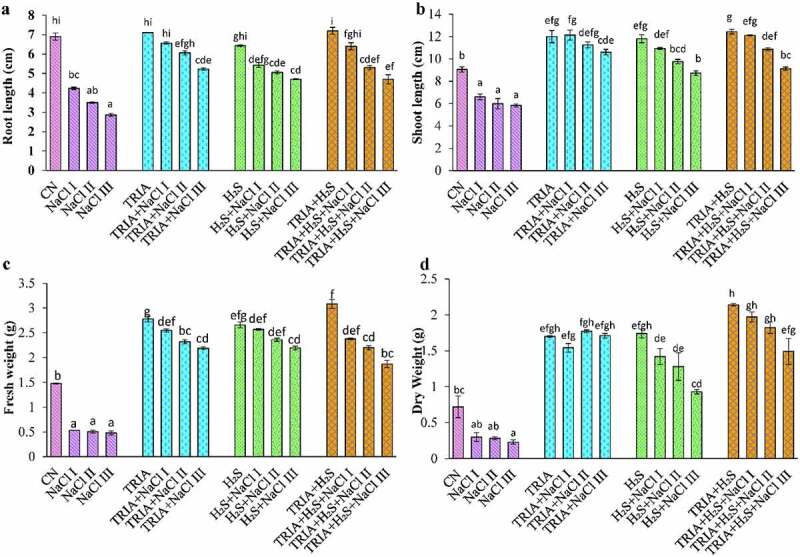
Effect of TRIA and H_2_S on root length (A), shoot length (B), fresh weight (C) and dry weight (D) in plants under salinity. Each number represents the mean of three replicates for each treatment level, as well as the standard error of the mean (SEM). Means inside a column separated by a distinct letter differ substantially at <p 0.05. CN- control; TRIA- Triacontanol; H_2_S- Hydrogen sulfide; NaCl I- 50 mM; NaCl II- 100 mM; NaCl III- 150 mM.

Salinity inhibited the plant growth factors, i.e., root length (Figure 1a), shoot length (Figure 1b), fresh weight (Figure 1c) and dry weight (Figure 1d) of brassica plants. However, exogenous treatment of TRIA and H_2_S alone or in combination substantially increased the plant growth parameters under stressed conditions. Root and shoot length reduced drastically at all three NaCl concentrations (50, 100, and 150 mM) by 39%, 49%, 56% and 27%, 33%, and 35%, respectively, when compared with CN plants. Individual application of TRIA and H_2_S significantly improved the root length. Only a 4% reduction in root length and a 32% increase in shoot length were observed in case of TRIA + NaCl I treatment with respect to CN plants. Combination of all three treatments, i.e., TRIA + H_2_S + NaCl I caused 4% in root length and 33% increase in shoot length, respectively, as compared to CN plants.

It was found from the results that treatment of NaCl resulted in reduced fresh and dry weight with 64%, 66%, 67%, and 58%, 61%, 68% in NaCl I, II, III concentrations, respectively, as compared to CN plants. Supplementation of TRIA and H_2_S alleviated salt stress in *B. juncea* plants. Fresh and dry weight were increased by 87% and 136% and 79% and 141%, respectively, in TRIA and H_2_S applied control plants, in comparison to CN plants. In case of TRIA + NaCl I treatment, an increase in (72%) fresh weight and (136%) dry weight was noticed, in comparison to CN plants. A combination of exogenous application of TRIA and H_2_S under salinity showed a decrease in fresh and dry weight by 25%, 28%, and 39% and 8%, 15%, and 30% at different concentrations of NaCl I, II, III, respectively, as compared to respective control plants.

### 3.2. TRIA and H_2_S-induced regulation of oxidative stress markers in salt-stressed plants

**Figure 2.**
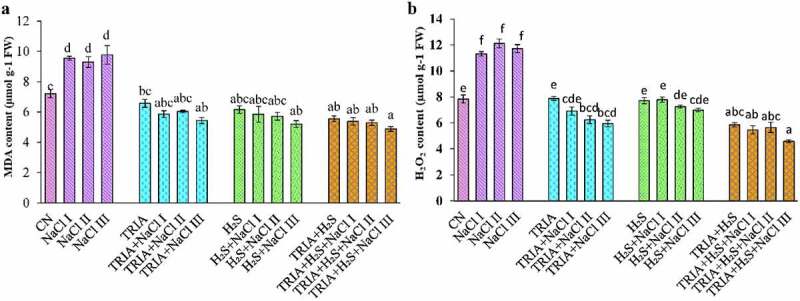
Effect of TRIA and H_2_S on MDA (a) and H_2_O_2_ (b) level in *B. juncea* plants under salinity. Each number represents the mean of three replicates for each treatment level, as well as the standard error of the mean (SEM). Means inside a column separated by a distinct letter differ substantially at <p 0.05. CN- control; TRIA- Triacontanol; H_2_S- Hydrogen sulfide; NaCl I- 50 mM; NaCl II- 100 mM; NaCl III- 150 mM.

Effect of salt stress on oxidative stress markers with respect to MDA and H_2_O_2_ content. NaCl I, II, and III concentrations caused 32%, 28%, and 35% elevation in the MDA content respectively, in comparison to CN plants (Figure 2a). However, individual application of TRIA and H_2_S at NaCl I, II, III concentration reduced the level of MDA by 10%, 8%, and 17%, and 5%, 7%, and 15% with respect to CN plants.

Level of H_2_O_2_ increased under all NaCl concentrations among which the highest H_2_O_2_ level was at NaCl III concentration. Combined as well as individual application of TRIA and H_2_S caused a significant decrease in content of H_2_O_2_ under stressed conditions with respect to NaCl treated plants. It was found that treatment of TRIA under NaCl III concentration showed 25% increase in comparison to NaCl III alone treated plants (Figure 2b). Combined treatment of TRIA and H_2_S under NaCl I and III concentration showed a significant decrease of 6% and 22% respectively.

### 3.3. Effect of TRIA and H_2_S on plant metabolites under salt stress

**Figure 3.**
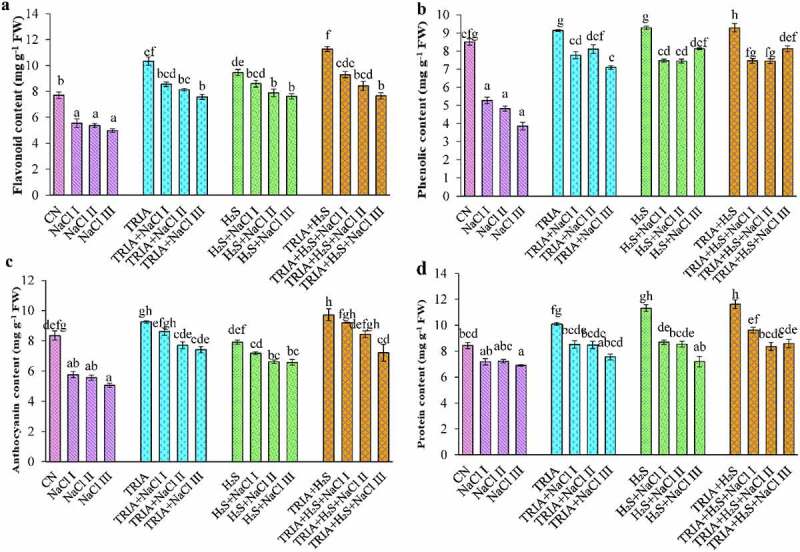
Effect of TRIA and H_2_S on flavonoid (a), phenolic (b), anthocyanin (c) and protein (d) content in *B. juncea* plants under salinity. Each number represents the mean of three replicates for each treatment level, as well as the standard error of the mean (SEM). Means inside a column separated by a distinct letter differ substantially at <p 0.05. CN- control; TRIA- Triacontanol; H_2_S- Hydrogen sulfide; NaCl I- 50 mM; NaCl II- 100 mM; NaCl III- 150 mM.

Flavonoid content drastically decreased at different concentrations of NaCl. It was found that NaCl-treated plants demonstrated a decline of 28%, 30%, and 35% at NaCl I, II, and III concentrations, respectively, when compared with CN plants (Figure 3A). Application of TRIA and H_2_S caused alleviation of salt stress by increasing flavonoid content with respect to NaCl plants. TRIA + NaCl I application caused only 17% reduction in flavonoid content in comparison to TRIA control plants. Combined treatment of TRIA and H_2_S showed an increase of 17%, 25%, and 32% in the flavonoid content at NaCl I, II, and III concentrations, respectively, as compared to CN plants.

Salinity reduced the phenolic content by 38%, 43%, and 54% at different concentration of NaCl I, II and III in comparison to control plants Supplementation of TRIA and H_2_S alone or in combination recovered NaCl-mediated decrease in phenolic content (Figure 3b). Increase in the level of phenolic content was observed at TRIA + H_2_S treatment by 22% as compared to CN plants. The combination of TRIA + H_2_S under NaCl III concentration showed the highest phenolic content as compared to TRIA+ H_2_S + NaCl I and TRIA + H_2_S + NaCl II treated plants.

Salt-stressed mustard plants showed a decline in anthocyanin content by 31%, 33%, and 39% at NaCl I, II and III concentrations, respectively, in comparison to CN plants. Present investigation revealed that supplementation of TRIA and H_2_S independently or in combination increased the anthocyanin content. Individual application of TRIA under NaCl I, II, and III concentrations showed a decline of 7%, 13%, and 26%, respectively, in anthocyanin content (Figure 3c) in comparison to CN plants. However, in comparison to the corresponding control plants, the individual administration of H_2_S at NaCl I resulted in a negligible rise of 9%, and a decrease of 16% and 8% at NaCl II and III concentrations, respectively.

### 3.4. TRIA and H_2_S-mediated protein content and antioxidative enzyme activity

Total protein content declined significantly under saline conditions in *B. juncea*. It decreased by 14%, 14%, and 18% at NaCl I, II, and III concentrations, respectively, in comparison to CN plants (Figure 3d). Whereas, exogenous treatment of TRIA, H_2_S and TRIA + H_2_S significantly alleviated salt stress in mustard plants. TRIA, H_2_S and TRIA + H_2_S treated control plants increased protein content by 19, 34, and 37%.

**Figure 4.**
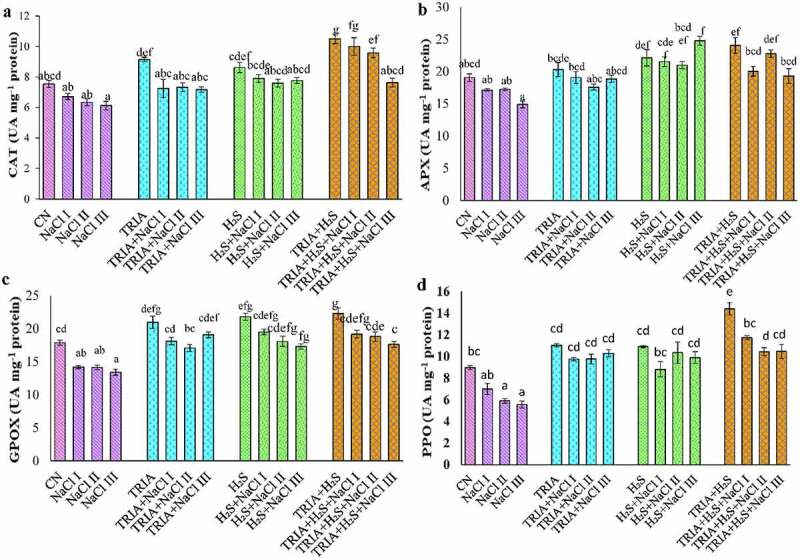
Effect of TRIA and H_2_S on CAT (a), APX (b), GPOX (c) and PPO (d) in *B. juncea* plants under salinity. Each number represents the mean of three replicates for each treatment level, as well as the standard error of the mean (SEM). Means inside a column separated by a distinct letter differ substantially at <p 0.05. CN- control; TRIA- Triacontanol; H_2_S- Hydrogen Sulfide; NaCl I- 50 mM; NaCl II- 100 mM; NaCl III- 150 mM.

Antioxidative enzymatic activities, i.e., CAT, APX, GPOX, and PPO, were evaluated. Under saline conditions, activity of enzyme CAT showed a prominent decrease in comparison to CN plants. Individual as well as the combined application of TRIA and H_2_S alone under non-saline conditions, CAT activity increased by 21%, 14% and 39%, (Figure 4a) as compared to CN plants. TRIA treatment under salinity increased the CAT activity in comparison to NaCl alone treated plants. Combined application of TRIA + H_2_S salt stress showed a maximum increase in the CAT activity at NaCl III concentration by 19% with respect to to CN plants.

The activity of APX enzyme was influenced by the application of TRIA and H_2_S under salt stress. Supplementation of TRIA caused elevation of 1% at NaCl I concentration while observed only a decrease 7% and 3% in case of NaCl II and III concentration in comparison to CN plants (Figure 4b). Combined application of TRIA and foliar applied H_2_S mitigated salinity by 16%, 5%, and 20% at NaCl I, II, and III respectively, in comparison to control plants, i.e., TRIA+H_2_S.

GPOX activity declined drastically under salt stress than CN plants. Whereas supplementation of TRIA and H_2_S raised in the level of GPOX as compared to CN plants. Maximum GPOX activity was examined at TRIA+H_2_S treatment followed by TRIA and H_2_S alone treated plants under unstressed conditions. Individual treatment of TRIA caused elevation in the GPOX activity by 1%, 4%, and 7% at NaCl I, II, and III, respectively, as compared to CN plants (Figure 4c). In TRIA + H_2_S plants under NaCl I stress, a 7% increase in the activity of GPOX was found in comparison to CN plants.

The PPO enzyme activity was reduced under salt stress with a maximum reduction of 41% at NaCl III concentration in comparison to CN plants. TRIA and H_2_S application, alone or in combination, raised PPO activity of *B. juncea* plants under salt stress (Figure 4d). Individual application of TRIA and H_2_S showed a substantial rise in PPO activity, in comparison to CN plants. Supplementation of TRIA and H_2_S individually showed a reduction of 12%, 12%, and 7% and 19%, 5% and 9% at NaCl I, II and III concentrations, respectively. Combined supplementation of TRIA and H_2_S under salt stress demonstrated maximum PPO activity at NaCl III concentration with 31% increase, in comparison to CN plants.

### 3.5. Effect of TRIA and H_2_S on non-enzymatic antioxidants of B. juncea under salt stress

**Figure 5.**
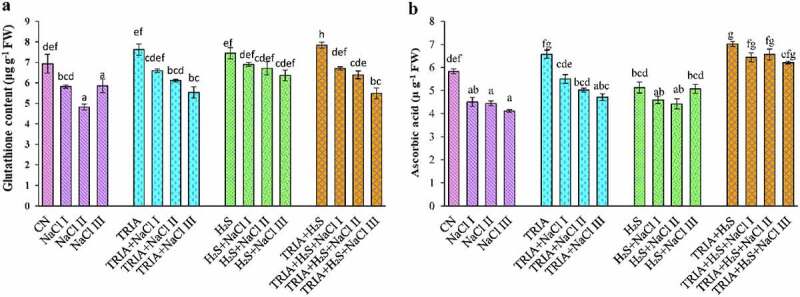
Effect of TRIA and H_2_S on glutathione (a) and ascorbic acid (b) content in *B. juncea* plants under salinity. Each number represents the mean of three replicates for each treatment level, as well as the standard error of the mean (SEM). Means inside a column separated by a distinct letter differ substantially at <p 0.05. CN- control; TRIA- Triacontanol; H_2_S- Hydrogen Sulfide; NaCl I- 50 mM; NaCl II- 100 mM; NaCl III- 150 mM.

The GSH content was found to highly decrease at NaCl III concentration by 30% in comparison to CN plants (Figure 5a). Individual as well as combined supplementation of TRIA and H_2_S increased the GSH level as compared to CN plants. Supplementation of TRIA ameliorated soil salinity by a decrease of 5.66%, 13.89%, and 19.21% at NaCl I, II and III concentrations. Individual application of H_2_S ameliorated soil salinity toxicity by a decrease of only 13%, 10% and 27% at NaCl I, II, and III concentrations in comparison to control plants, i.e., H_2_S alone. Combined supplementation of TRIA and H_2_S improved the GSH content by 13% at NaCl I concentration, as compared to CN plants.

Ascorbic acid amount was lowered in the case of salt-treated plants. However, application of TRIA and H_2_S separately or in combination improved the AsA amount under salt stress (Figure 5b). Maximum content was observed at TRIA + H_2_S treated plants. In case of combined treatment of TRIA and H_2_S, only a decrease of 6%, 8%, and 11% was observed at NaCl I, II, and III concentrations in relative to their control plants, i.e., TRIA + H_2_S.

### 3.6. Principal component and correlation analysis

**Figure 6.**
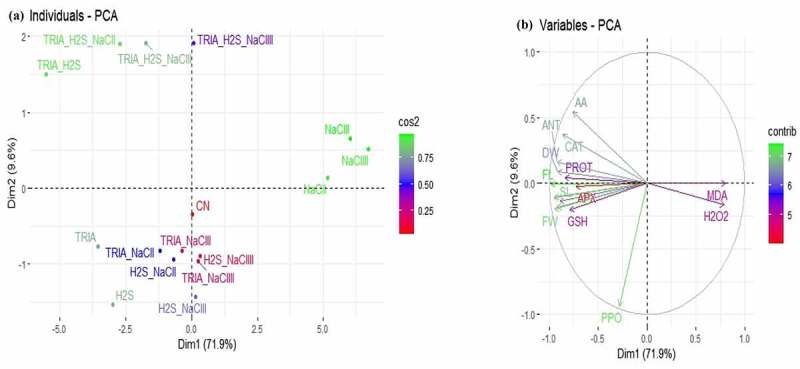
Principal component analysis (PCA) of (a) individual treatments by PCA and (b) diverse analyzed parameters of Brassica plants under NaCl stress. (A) Score plot indicates the separation of treatments of TRIA, NaCl, H_2_S, TRIA+ H_2_S, TRIA + NaCl, H_2_S + NaCl and TRIA + H_2_S + NaCl.

Abbreviations: Triacontanol (TRIA); hydrogen sulphide (H_2_S); sodium chloride (NaCl); root length (RL); shoot length (SL); fresh weight (FW); dry weight (DW); malondialdehyde (MDA); hydrogen peroxide (H_2_O_2_); flavonoid (FL); protein (PROT); phenolic (PHEN); anthocyanin (ANT); catalase (CAT); ascorbate peroxidase (APX); guaiacol peroxidase (GPOX); polyphenol oxidase (PPO); glutathione (GSH); ascorbic acid (AA).

To evaluate the impact of TRIA and H_2_S treatments on the explored traits of *Brassica* plants, the loading and score diagrams of PCA were implemented (Figure 6). The initial two components, i.e., Dim1 (PC1, 71.9%) and Dim2 (PC2, 9.6%) exhibited the highest participation and represented 80% of variance was noticed. Pertaining to TRIA, H_2_S, and NaCl application, similar treatments at various levels/combinations were grouped nearby. On the other hand, the first two elements effectively separated were various therapies (Figure 6a). This division of the diverse treatments obviously suggested that the TRIA and H_2_S treatments under NaCl stress had a strong ameliorative effect on the examined traits of brassica plants than CN. The first set of the variables of PCA (PC1), is found to be positively correlated with most of the variables such as root length, shoot length, fresh weight, dry weight, flavonoid, protein, phenolic, anthocyanin, catalase, ascorbate peroxidase, guaiacol peroxidase, polyphenol oxidase, glutathione, and ascorbic acid (Figure 6b). In response to this, worthy correlation between PC1 variables (H_2_O_2_ and MDA) was found to be in line up with PC2 (Figure 6b).

**Figure 7.**
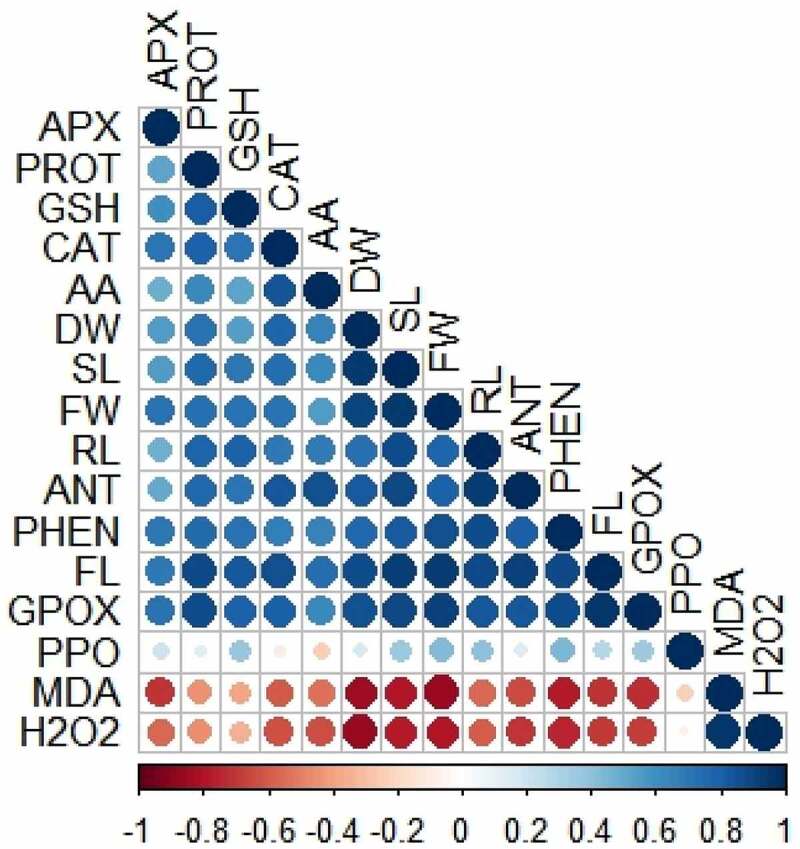
Pearson’s correlation analysis between diverse analyzed parameters of brassica plants under NaCl stress. Blue and brownish colors indicate positive and negative correlation, correspondingly. Abbreviations: Root length (RL); shoot length (SL); fresh weight (FW); dry weight (DW); malondialdehyde (MDA); hydrogen peroxide (H_2_O_2_); flavonoid (FL); protein (PROT); phenolic (PHEN); anthocyanin (ANT); catalase (CAT); ascorbate peroxidase (APX); guaiacol peroxidase (GPOX); polyphenol oxidase (PPO); glutathione (GSH); ascorbic acid (AA).

A Pearson’s correlation analysis was carried out between diverse analyzed traits of brassica plants (Figure 7). The analysis exhibited that H_2_O_2_ and MDA showed negative correlation with other characteristics like root length, shoot length, fresh weight, dry weight, flavonoid, protein, phenolic, anthocyanin, catalase, ascorbate peroxidase, guaiacol peroxidase, polyphenol oxidase, glutathione, and ascorbic acid. In contrast, all other traits showed a strong positive correlation (Figure 7). This correlation characterizes relationship between growth attributes, metabolites, antioxidant defense systems in *Brassica* plants.

## 4. Discussion

In the present research, it was examined that salt stress had a major impact on growth and biomass of *B. juncea* plants. Salt stress induces water and ionic imbalances in plants due to the presence of toxic ions. Harmful impact of salt stress on growth factors was observed in different studies.^3,4^ However, the use of TRIA and H_2_S separately and in combination reduced the salinity stress and enhanced the development of salt-stressed *B. juncea* plants. Zhang, Hu^44^ found that TRIA boosted the root length, shoot length, and plant biomass in *Zea mays* under salt stress. TRIA application improved plant tolerance against salt stress by augmentation in the cell wall extension and cell division. Various studies have reported that TRIA application at various stages of growth, i.e., vegetative, flowering, and podding stages were more efficient in enhancing crop growth, yield and production as it exert essential effect equally at different stages of growth.^45^ It has been suggested that increased nutrient intake, improved rate of photosynthesis, and increased mobilization of reserve materials caused by the application of TRIA raised plant growth and final productivity.

Moreover, TRIA is recognized to boost growth, quality, content and productivity of numerous crops.^46–48^ Apart from TRIA, H_2_Sinfluence wide range of physiological and developing activities in plants, such as growth, development of lateral root formation, seed germination,^44^ lateral root formation,^49^ against numerous biotic and abiotic stresses. NaHS application in low concentrations affect activity of various secondary metabolites, osmolytes as well as various antioxidant and non-antioxidant enzymes in grapevine (*Vitis vinifera* L.)^26^.

Phenolics are known for their excellent antioxidant properties that play an essential role in eliminating singlet oxygen and which improve the tolerance toward salinity.^50^ Due to their function in chelating and scavenging, phenolic chemicals reduce salt stress by stabilizing cellular membrane. Therefore, these compounds experience various adaptive mechanisms in response to stressful situations.^51^ Recently it was found that TRIA and H_2_S application under salt stress increased the level of flavonoids, phenolics and anthocyanins. Phenolic compounds produced by phenylpropanoid or shikimic acid pathway play an important role alleviating different kind of stresses due to its antioxidant properties.^52^ The potential and accessibility of radicals in the termination reaction determine their capacity to function as antioxidants.^53,54^

Flavonoids are another category of plant phenolics that perform a variety of activities in the plant system and decrease the detrimental outcome of abiotic stresses.^55^ Recently, it was found that TRIA application enhanced the flavonoid content in *B. juncea* plants under salinity stress. Similarly, it was found that exogenous application of TRIA under abiotic stress conditions increased the flavonoid content in wheat (*Triticum aestivum*).^24^ Furthermore, it was found that TRIA application removed harmful effect of metal stress in the case of different varieties of wheat.^24^ Kumaravelu et al.^56^ discovered that under either control or stressed conditions, soaking of seeds without TRIA had no effect on phenolics content. However, green gram seeds with TRIA application showed an increase in phenolics content under both normal and stressful conditions. In the case of fruits, the levels of flavonoid and phenols increased rapidly after treatment with H_2_S, which would delay fruit senescence and decay.^57^

Lipid peroxidation, which is brought on by salt stress, is frequently utilized as a sign of salt-related oxidative membrane damage.^3,58^ Malondialdehyde buildup from membrane lipid peroxidation, is a sign of membrane deterioration in salt-stressed cells.^59^ Due to lipooxygenases or ROS, membrane lipids become peroxidized.^60^ Parallel to this observation, it was found that TRIA has anti-inflammatory and powerful antioxidizing properties as it causes lipid peroxidation in membrane lipids due to its inhibitory effect.^61–63^ Application of TRIA repressed lipid peroxidation in spinach (*Spinacia oleracea* L.)^62^ and in leaves of *Arachis hypogaea* L. which in turn improved integrity of membranes.

Leakage of electron, and content of MDA and H_2_O_2_ markedly lowered due to supplementation of NaHS, H_2_S donor when applied on strawberry plants which were suffering from osmotic stress. Likewise, it was found that H_2_S administration mitigates the adverse effects of salt stress in plants by improving antioxidant mechanism, playing crucial role in reduction of H_2_O_2_ and MDA level.^64^ Complex antioxidant defense systems like SOD, POD, and CAT pathways are activated which provide resistance against numerous abiotic stresses. Hence, our study suggests that treatment of TRIA + NaHS reduces the content of H_2_O_2_ and MDA by antioxidant enzymatic activity in *B. juncea*, confirming that pre-treatment of TRIA and H_2_S mitigate salt stress by activating antioxidant enzymatic activity.

ROS generation in plants affects plant growth by causing damage of numerous cellular components^3,12^. Overproduction of ROS in plants causes damage to various bimolecular structure like lipids, proteins, and nucleic acids^3,12,54,65^. Therefore, cellular ROS balance must be maintained by regulating their production and scavenging, which is maintained by antioxidant defense system such as SOD, CAT, GPX, POD, APX, and GR, and non-enzymatic antioxidants, i.e., AsA and GSH.^54,66,67^ These findings further suggested pre-treatment of NaHS improved the antioxidant enzymatic activity.^68^ TRIA application alone or in combination enhanced the activity of enzymatic and non-enzymatic antioxidants. TRIA application when applied in the form of foliar spray has been reported to influence numerous metabolic processes by activating numerous enzymes.^69^ Praveen et al.^25^ found that the improvement in growth brought on by TRIA may have resulted from the way it affected the function of POD and other antioxidant enzymes under salt stress. Altering the redox state via antioxidant enzymes like SOD and CAT reduced the As-induced oxidative stress in coriander plants by supplementation of TRIA^70^. Moreover, H_2_S mitigates oxidative stress due to salinity by ROS generation in plants and by stimulating the enzymatic and non-enzymatic antioxidants. Stress caused due to salinity was alleviated by mediating the expression and activity of antioxidant enzymes like SOD, POD, CAT, APX, GR, PPO, and GPOX.^71–74^ Application of NaHS greatly boosted the activities of important enzymes i.e., L-galactose dehydrogenase (GalDH), and MDHAR. Saline-alkali stress caused oxidative damage to naked oats; however, the addition of H_2_S modified the activity of key enzymes such as AO, APX, and GR^75^ which play an essential role in H_2_O_2_ detoxification are CAT and APX. Additionally, it is understood that balance of SOD and H_2_O_2_ scavenging enzymes is essential in determining buildup of O_2_ and H_2_O_2_ in plants^75^. The first line of defense against ROS is SOD.^67^ Exogenous application of TRIA considerably alleviated the salinity stress in wheat plants by boosting the SOD activity.^76^ The process of CAT involves changing H_2_O_2_ into H_2_O and O_2_. By displacing Fe from the CAT active center, cadmium prevents the enzyme from working.^77^ Similar results were obtained by^78^ in *Ocimum basilicum* L. under chilling stress, who found that foliar application of TRIA enhanced CAT activity. Numerous enzymes are activated by TRIA, which are involved in carrying out important metabolic pathways, including peroxidase, rubisco, dehydrogenase, and Ca^2+/^Mg^2+^ dependent ATPases.^79,80^ Therefore, the antioxidant enzymatic system alleviates stress condition by ROS detoxification in plants of *B. juncea*.

The combined action of antioxidant mechanisms reduces the oxidative damage caused by salt stress. These include ascorbate and glutathione. Under abiotic stress, several physiological processes result in the production of superoxide radicals.^81^ Plant organelles that contain glutathione operate as buffers, allowing the plant to control redox equilibrium and lessen the impact of abiotic stressors.^82^ It results in ROS detoxification, metabolite conjugation, xenobiotic detoxification, and signaling action that sets off adaptive responses in stressful situations.^83^ Researchers have discovered a connection between elevated antioxidant enzyme activity and improved production of biomass which resist different stresses like drought stress in rice.^84^ It was reported that H_2_S mitigated negative effect by enhancing the activities of AsA and GSH. H_2_S acted as upstream in regulating the activity of AsA-GSH cycle in wheat seedlings.^85^ Furthermore, it was found that both AsA-GSH systems manage redox metabolism in maize by regulating of AsA-GSH cycle which play essential role in ROS homeostasis.^86^

## 5. Conclusion

**Figure 8.**
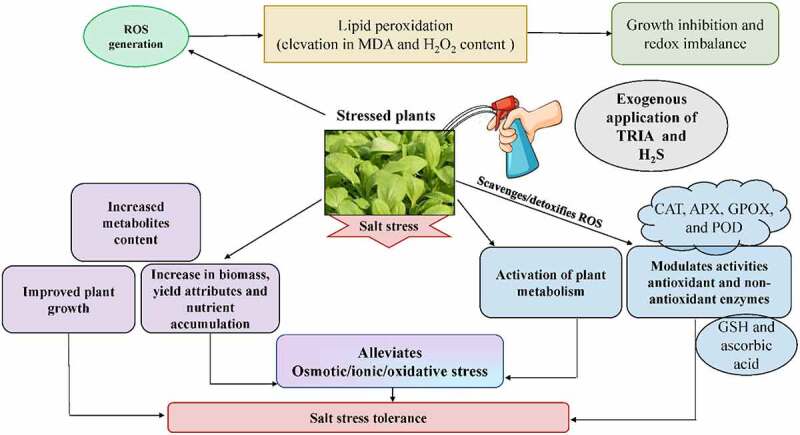
Triacontanol and hydrogen sulfide-mediated salt tolerance in *B. juncea*.

This study depicted that salt stress triggered a decline in plant growth and biomass. To counteract salt stress, TRIA and H_2_S application, individually or in combination, activated antioxidant defense system to reduce ROS levels in plants of *B. juncea* (Figure 8). As a result, exogenous application of TRIA and H_2_S, particularly in combination, can be recommended as a cost-effective and environmentally acceptable method to reduce salinity on a commercial scale.
